# Assessing the Bowing Technique in Violin Beginners Using MIMU and Optical Proximity Sensors: A Feasibility Study

**DOI:** 10.3390/s21175817

**Published:** 2021-08-29

**Authors:** Cecilia Provenzale, Nicola Di Stefano, Alessia Noccaro, Fabrizio Taffoni

**Affiliations:** 1 CREO Lab–Advanced Robotics and Human-Centred Technologies, Università Campus Bio-Medico di Roma, 00128 Rome, Italy; c.provenzale@unicampus.it; 2 Department of Philosophy and Cultural Heritage, Ca’ Foscari University of Venice, 30123 Venice, Italy; n.distefano@unicampus.it; 3 Research Unit of Philosophy of Science and Human Development, Campus Bio-Medico University, 00128 Rome, Italy; 4 Research Unit of Neurophysiology and Neuroengineering of Human-Technology Interaction (NeXTlab), Università Campus Bio-Medico di Roma, 00128 Rome, Italy; a.noccaro@unicampus.it

**Keywords:** human movement, motor control, motor learning, feedback, MIMU, variability, behavior

## Abstract

Bowing is the fundamental motor action responsible for sound production in violin playing. A lot of effort is required to control such a complex technique, especially at the beginning of violin training, also due to a lack of quantitative assessments of bowing movements. Here, we present magneto-inertial measurement units (MIMUs) and an optical sensor interface for the real-time monitoring of the fundamental parameters of bowing. Two MIMUs and a sound recorder were used to estimate the bow orientation and acquire sounds. An optical motion capture system was used as the gold standard for comparison. Four optical sensors positioned on the bow stick measured the stick–hair distance. During a pilot test, a musician was asked to perform strokes using different sections of the bow at different paces. Distance data were used to train two classifiers, a linear discriminant (LD) classifier and a decision tree (DT) classifier, to estimate the bow section used. The DT classifier reached the best classification accuracy (94.2%). Larger data analysis on nine violin beginners showed that the orientation error was less than 2°; the bow tilt correlated with the audio information $\left(r\left(134\right)=-0.973,\;95\%\;\mathrm{CI}\;\left[-0.981,-0.962\right],\;\;p<0.001\right)$. The results confirmed that the interface provides reliable information on the bowing technique that might improve the learning performance of violin beginners.

## 1. Introduction

A violin is one of the most complex musical instruments to learn. Its structure forces the trainee to maintain unnatural body postures for long periods. Moreover, since producing pleasant sounds is a hard task that requires a precise model of the instrument to properly interact with it, the initial phase of training is not rewarding for practitioners in terms of auditory feedback.

Despite the different methods that are adopted to teach the violin, it is common to start training the fundamentals of bowing techniques with a series of basic exercises focused on elementary motor skills. In particular, the first year of study is almost entirely dedicated to learning how to perform bowing on open strings (i.e., without pressing strings on the neck with the left hand) in a natural, fluid, and smooth way. Such bowing gestures require the development of precise sensorimotor control both in time and space domains. It has been estimated that 10,000 h of training is needed to bring fine sensorimotor control to a professional level [1].

In the first phase of teaching, teachers hold the arms of the students and guide their gestures so they perform the correct movement. In this way, with practice and the teachers’ feedback, beginners gradually learn to automatically perform the correct movement. Learning musical instruments is strongly based on such teacher–student interaction, where teachers show how to perform a specific exercise and students attempt to imitate their performance. This process requires time and the constant supervision of the teacher. To reduce learning time, technological solutions might be developed to provide students with reliable feedback about their movement, even when the teacher is not physically present. Such technologies should allow monitoring of the factors that have the maximum influence on the quality of the sound emitted [2,3]: (i) the bow section, i.e., the transversal bow portion in direct contact with the string; (ii) the bow–bridge distance, i.e., the distance between the bridge and the point of the string in contact with the bow hair; (iii) the bow velocity; and (iv) the bow force, i.e., the force applied by the musician through the bow on the string. Although each of these parameters influences sound quality, in the initial stages of training, trainees focus most on controlling bow orientation with respect to the violin strings to keep the bow–bridge distance constant and play one string at a time. Such skill can be worked on by dividing the bow into three parts: the lower section (closest to the hand), the middle section, and the upper section (the most distal part of the bow).

A number of studies have applied technologies to the analysis of bowing movements. For example, the bow–bridge distance is measured in [4] by using a resistive voltage divider. In [5,6], the authors propose the use of a resistive strip placed along the bow stick. Two square waves at different frequencies are generated at the opposite ends of the strips. An electrode antenna, placed behind the bridge, receives through capacitive coupling the combined signal coming from the bow and sends it to a remote unit. The latter separates the two signals from each other. The proportion of the two coupled frequencies varies with the transverse position of the bow. Bow force is generally measured by using strain gauges, usually placed on the bow body [4,5,7]. Pardue et al. [8,9] use four optical sensors, placed on the bow stick at specific distances from the frog, to measure the pressure exerted on the string from the bow, as well as to identify the position of the contact point between the bow hair and the string. In [5,10], a wireless measurement of the acceleration of the bow is carried out by means of two accelerometers mounted on the frog. In [11,12], the violin and bow poses are measured by means of an optical marker system. Van der Linden et al. [13] combine motion capture based on optical markers with a wearable system to provide vibrotactile feedback (music jacket). This allows children of different ages to exploit real-time vibrotactile feedback delivered to the upper limb to learn the bowing technique. From a technical perspective, the main limitation of the above approaches is the system used to measure bowing techniques. In particular, while optical markers guarantee a high level of accuracy, the small dimensions of the objects involved (i.e., the violin and the bow) limit the possibility of opportunely placing the marker. Moreover, these systems require several cameras to avoid problems of marker occlusion and need to strongly structure the environment with high-cost technology.

While most systems allow for monitoring different aspects of bowing, only a few of them [5,13] are specifically developed for teaching, and apparently none of them can be effectively used at home with a low-cost technology. Table 1 summarizes the technologies used. Each technology is assessed in terms of the number of variables it allows measurement of and with respect to a set of usability parameters we derived from the system usability scale [14].

Technological solutions that might be exploited for developing a real-time interface for learning in violin-playing scenarios should be cheap; usable in minimally structured environments, e.g., at home; and non-invasive. For these reasons, based on Table 1, we carry out a feasibility study on the use of use magneto-inertial measurement units (MIMUs) and optical proximity sensors for assessing bow orientation and bow section, respectively. Indeed, MIMUs embed inertial accelerometers and gyroscopes as well as magnetometers. This set of sensors allows easy reconstruction of the orientation solely relying upon gravitational and geomagnetic fields, which are present everywhere on earth, without the need of other sources of fields (sourceless). The optical proximity sensors are usually composed of a transmitting module and a receiving module. They are typically developed with an LED emitting on a specific bandwidth coupled with a phototransistor. In reflective configuration, the LED and the phototransistor are mounted side by side in the same plastic small case (usually a square chip a few millimeters long and wide and 1–2 mm in height) and can be easily used to measure the change in the distance of the objects placed in front of the case. Such technologies are non-invasive and cheap and can be easily embedded into a bow and a violin. The present interface could foster the learning process by assisting violin beginners during their training as it provides the students with feedback, even when the teacher is not physically present.

## 2. Materials and Methods

During the first year of training, violin beginners learn to control two fundamental parameters of the bowing technique that strongly affect the quality of the sound produced: the orientation of the bow with respect to the violin body and the contact point between the bow and the string. To correctly perform bowing movements, the bow trajectory should be maintained perpendicular to the violin strings during the whole movement. Moreover, knowing the contact point between the bow and strings allows the learner to master the different sections of the bow (i.e., upper, middle, and lower), each one having different mechanical properties that allow for different usages of the bow (e.g., different bow strokes).

**Figure 1:**
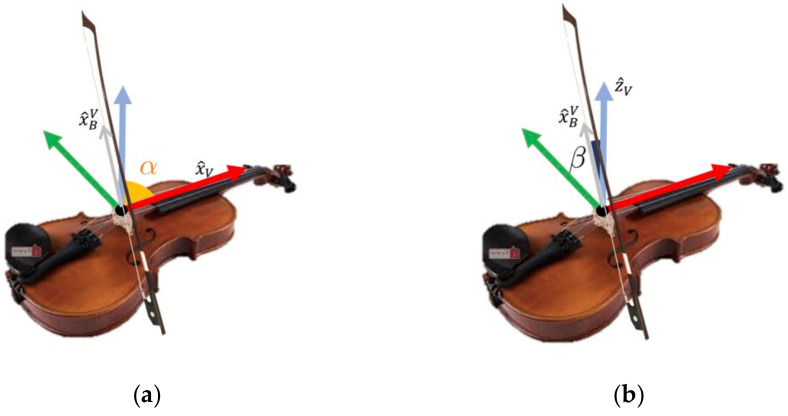
(**a**) 𝛼 is the angle between the bow hair and the violin string; (**b**) 𝛽 is the angle between the bow hair and the axis normal to the violin soundboard ($\hat{z_{V}\;}$). $\hat{x_{V}\;}$ and $\hat{z_{V}\;}$ are, respectively, the x-unit vector and the z-unit vector of the violin triad. ${\hat{x}}_{B}^{V}$ is the projection of the x-unit vector of the bow reference frame in the violin reference frame, as described in Equation (1).

To assess bow orientation, we considered two angles: the one between the bow hair and the violin string (i.e., the α angle, Figure 1) and the angle between the bow hair and the axis normal to the violin soundboard (i.e., the β angle, Figure 1). The α angle should be kept at approximately 90° to maintain the bow–bridge distance constant during the execution of a stroke, thus controlling the amplitude of the string’s vibration [4]. A proper tilt of the bow (the β angle or the bow tilt) guarantees that only the effective string will be played.

**Figure 2:**
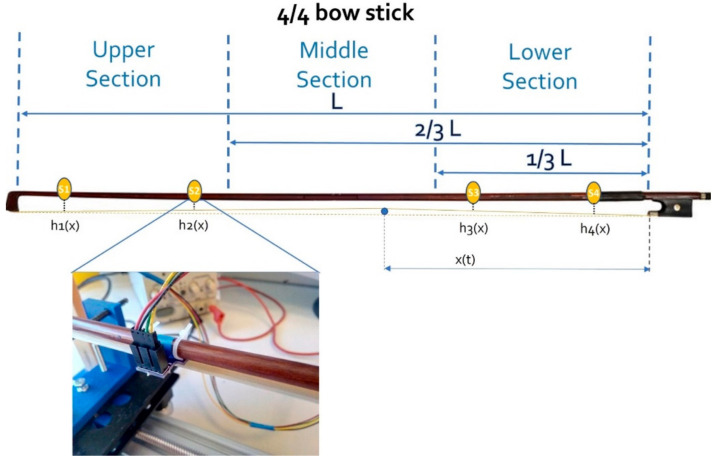
Sections of the bow and position of the optical sensors (see Section 2.2). The sensors from S4 to S1 are placed, respectively, about L/14, 3/13 L, 3/4 L, and 24/25 L from the frog using a cable tie (see the detailed view reported for sensor S2). The blue dot represents the section of the string in contact with the bow hair. Sensor outcomes depend on the position (x(t)) of the contact point.

Concerning the contact point between the bow and strings, we divided the bow length into three sections (Figure 2): lower, the section closest to the frog; middle, in which the equilibrium point of the bow typically falls; and upper, the most distal part of the bow. We considered such division in line with technical exercises typically performed during the first year of violin training [15]. Indeed, during training, students are often requested to perform the same exercise using upper, middle, and lower sections. Moreover, some advanced techniques, such as the *balzato* and *saltellato*, are performed exclusively using specific sections of the bow due to their mechanical properties.

Since our aim was to demonstrate the feasibility of using a new interface in a specific application, we decided to involve a few healthy adult volunteers to simulate the final application scenarios, as explained in the following sections. When designing protocols for assessing the feasibility of a technology to be used with human subjects, it is of paramount importance to select commercial devices approved for use with humans, thus making it easier to obtain the approval of the institutional review board. In case it is necessary to integrate some ad hoc solution, it is also important to provide all technical information about safety, allowing the ethical committee to make an informed decision. This study was preliminarily approved by the ethics committee of the Università Campus Bio-Medico di Roma, and informed consent was obtained from all subjects involved in the study.

### 2.1. Measuring Bow–Violin Orientation

**Figure 3:**
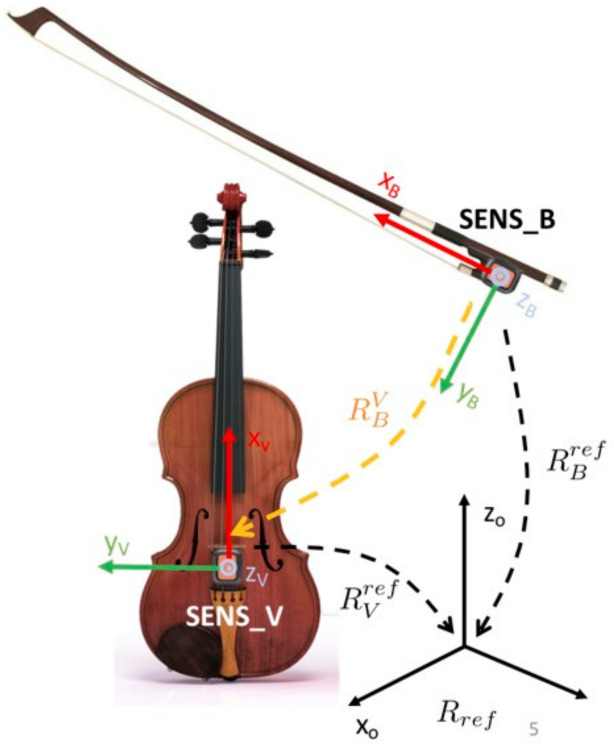
Arrangement of sensors on the violin and on the bow. $R_{V}^{ref}$ expresses the orientation of the violin with respect to the reference triad, represented in black. $R_{B}^{ref}$ expresses the orientation of the bow with respect to the reference triad, represented in black.

To estimate the orientation of the bow with respect to the violin body, we used two XsensDots produced by Xsens Inc, small and light enough to be easily embedded on the violin and on the bow. One sensor was mounted on the violin body between the tailpiece and the bridge, with the box edge in contact and aligned with the bridge surface, and the *x*-axis pointing in the direction of the strings (see SENS_V in Figure 3). The other one was positioned on the frog, with its *x*-axis pointing in the direction of the bow hair (i.e., SENS_B in Figure 3). Sensors were configured in data fusion mode to return their orientation with respect to a local earth reference frame. With sensors in the XsensDots docking station, we performed a heading reset operation to guarantee the alignment of the local earth reference frame of each sensor and compensate for a possible noise source. We labeled this common reference frame $R_{ref}$. We expressed the orientation of the bow with respect to the violin body in terms of the orientation matrix ($R_{B}^{V}$) as

(1) $$R_{B}^{V}=\left[\begin{array}{ccc} {\hat{x}}_{B}^{V} & {\hat{y}}_{B}^{V} & {\hat{z}}_{B}^{V} \end{array}\right],$$

where the columns of the matrix are the projections of the unit vectors of the bow reference frame in the violin reference frame. This matrix was derived from the orientation of the sensors with respect to the common reference frame, as in Figure 3:

(2) $$R_{B}^{V}={\left(R_{V}^{ref}\right)}^{T}*\;R_{B}^{ref}=R_{ref}^{V}\;R_{B}^{ref}$$

Knowing the relative orientation of the bow with respect to the violin body (1–2), it is possible to estimate the 𝛼 and 𝛽 angles as follows (Figure 1):

(3) $$\left\|{\hat{x}}_{B}^{V}\times {\hat{x}}_{V}\right\|=\|{\hat{x}}_{B}^{V}\|\;\|{\hat{x}}_{V}\|\;\sin\alpha \equiv \sin\alpha$$

(4) $${\hat{x}}_{B}^{V}\;\cdot \;{\hat{x}}_{V}=\|{\hat{x}}_{B}^{V}\|\;\|{\hat{x}}_{V}\|\;\cos\alpha \equiv \cos\alpha$$

(5) $$\alpha =\mathrm{arctg}\left(\frac{\left\|{\hat{x}}_{B}^{V}\times {\hat{x}}_{V}\right\|}{{\hat{x}}_{B}^{V}\;\cdot \;{\hat{x}}_{V}}\right)$$

(6) $$\left\|{\hat{x}}_{B}^{V}\times {\hat{z}}_{V}\right\|=\|{\hat{x}}_{B}^{V}\|\;\|{\hat{z}}_{V}\|\;\sin\beta$$

(7) $${\hat{x}}_{B}^{V}\;\cdot \;{\hat{z}}_{V}=\|{\hat{x}}_{B}^{V}\|\;\|{\hat{z}}_{V}\|\;\cos\beta$$

(8) $$\beta =\mathrm{arctg}\left(\frac{\left\|{\hat{x}}_{B}^{V}\times {\hat{z}}_{V}\right\|}{{\hat{x}}_{B}^{V}\;\cdot \;{\hat{z}}_{V}}\right)$$

**Figure 4:**
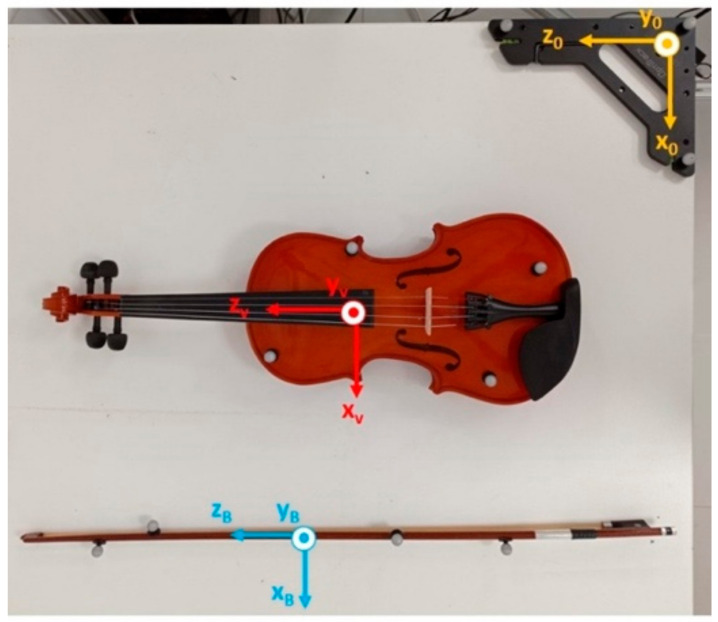
Motive triads on the violin and the bow. The triad in yellow is the Motive Optitrack reference triad.

To validate the use of MIMUs in estimating bowing angles, we decided to compare the orientations obtained using this technology with the ones obtained using an optical motion capture system (Motive Optitrack, Natural Point Inc., Corvallis, OR, USA) used as the gold standard. Figure 4 shows the reference triads defined for the bow and the violin, as well as the global reference frame of Motive Optitrack (in yellow). We used eight passive reflective markers, four on the violin and four on the bow, placed in a non-aligned way. The system was equipped with six cameras configured with a sample frequency of 120 Hz. The rotation matrix $R_{B}^{V}$, which expresses the bow orientation with respect to the violin, was obtained as in Equation (2) using the orientation data derived from the optical motion capture system. Data from XsensDots and Motive Optitrack were synched with a manual procedure: at the beginning of each acquisition, the subject placed the bow on the violin strings and rotated it around the *z*-axis of the SENS_B reference frame (Figure 3) three times. This operation produced three peaks in the 𝛽 angle traces, which were used to align the two data sources. Additionally, the sound produced was registered by a digital audio recorder (H4nPro, Zoom). Data were obtained from 9 violin beginners (2 females and 7 males) aged 33.4±7.7 years (mean ± SD). After the manual synch, subjects were requested to assume the correct pose, with the bow approximately perpendicular to the strings and parallel to the soundboard at the beginning of each trial. They had to maintain this initial reference position for about 5 s. The subjects were then requested to perform a simple exercise: to repeat twice a full bow stroke on each open string (starting from the tip, passing through the frog, and returning to the tip), beginning with the E string.

**Figure 5:**
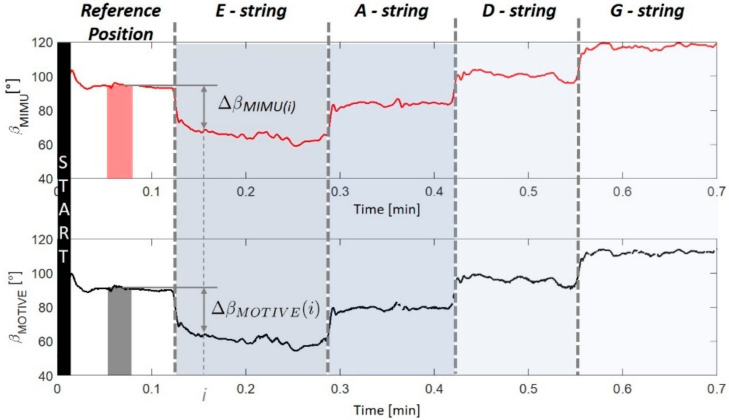
Example of computation of angular displacement Δβ with respect to the reference position.

To compensate the misalignment between reference frames of XsensDots and Motive Optitrack, the bow–violin orientation (α and β angles) measured with the two systems was referred to the initial reference position assumed at the beginning of the open string exercise. Figure 5 reports an example of computation for the β angle. The angular displacement with respect to the reference position was defined as

$$\Delta \alpha =\alpha -\alpha_{0}\;\Delta \beta =\beta -\beta_{0}$$

Here, $\alpha_{0}\;\mathrm{and}\;\beta_{0}$ are the 1 s averages of the α angle and the β angle, respectively, which are measured in the central portion of the reference position interval. The orientation error was measured as the absolute value of the difference between the angular displacement estimated with the XsenDots sensors and the one obtained with Motive Optitrack. This value was subsequently averaged for each string in order to obtain a mean absolute error (MAE). We used Matlab R2021a for the analysis.

The estimation of the orientation angle between the bow and the violin also helps to derive useful information about the actual string played. In fact, since the same note (except for the low G) can be obtained by playing different strings with different fingerings, playing a specific note on a string requires the bow to be properly tilted. Learning how to tilt the bow in order to play a specific string is a fundamental skill to be acquired by beginners. Open string exercises are designed properly to practice this skill: students have to progressively reduce the tilt angle moving from the E to the G string. We averaged the β angle measured during each stroke, thus obtaining 16 repeated measures for each subject (4 measures per string). In order to verify the relation between the tilt angle (dependent variable) and the string frequency (independent variable) we performed a repeated measure correlation between them using the R software, Vienna, Austria, ver. 4.1.1 [16].

### 2.2. Estimating the Bow Section

**Figure 6:**
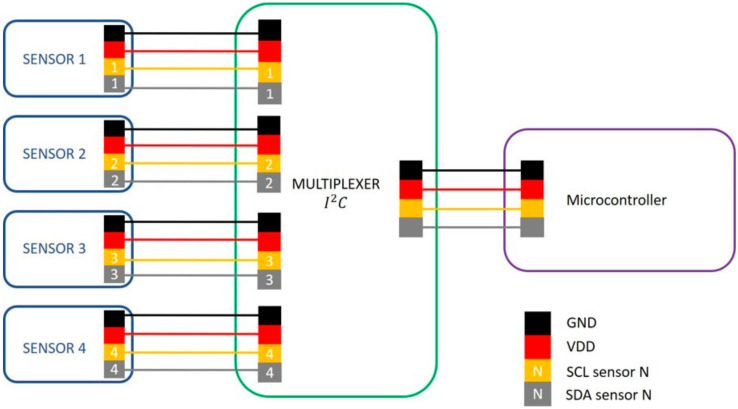
Communication architecture to manage data from optical proximity sensors. Because VCNL4040 sensors are provided with only one *I*^2^*C* address, an *I*^2^*C* multiplexer was added to allow the contemporary use of more than one sensor on the same bus.

We mounted four optical proximity sensors (VCNL4040, Vishay) on a 4/4 bow stick (L = 650 mm) to measure the distance between the bow hair and the stick. This distance ($h_{i}\left(x\right)$ with *i* as the sensor index, varying between 1 and 4) depends on the position of the sensors on the stick and the contact point between the bow hair and the violin string (x, Figure 2). Each sensor was calibrated using a micrometric screw (Newport M-460P Series) to finely tune the distance. The sensors, at the $I^{2}C$ interface, were connected to a control unit (PIC16F887, Microchip Technology Inc., Chandler, AZ, USA) by means of a multiplexer (PCA9548A, Texas Instruments) and sampled at 100 Hz (Figure 6). We placed sensors 45, 151, 485, and 624 mm from the frog (Figure 2), as in [8,9], and firmly fixed them with a cable tie.

In a pilot test, we asked an expert musician to perform 30 strokes in each section of the bow. The musician had to repeat the exercise at three different tempos for each string: slow (i.e., 60 bpm), medium (i.e., 80 bpm), and fast (i.e., 100 bpm). Section limits were clearly marked with a tape placed on the bow stick, while the velocity was paced by a metronome (see Supplementary Materials Video S1). Overall, 1080 bowings were performed: 30 (strokes) × 3 (speeds) × 3 (sections) × 4 (strings). We also acquired the bow stick–hair distances even when the bow was not in contact with the string. Because no actions were performed in this condition, we terminated the acquisition when 30 beats were counted in the three velocity modalities. Proximity data were used to train a classifier for identifying the section of the bow used. Among all the possible classifiers, we selected the decision tree (DT) and the linear discriminant (LD), mainly for their fast prediction speed and small memory usage. The DT classifier uses a tree structure from the root (beginning) node down to a leaf node. The leaf node contains the response. To predict a response, the algorithm starts from the top node, and at each decision, it checks the values of the predictors to decide which branch to follow. When the branches reach a leaf node, data are classified. In particular, the DT classifier used in this work allows a maximum of 100 splits [17,18]. Because our aim was to provide online support, we carefully tested the most efficient way to feed the classifier in order to provide timely feedback. We used the distances measured by the four sensors. Data were normalized with respect to the full scale of the sensors and were divided by using a moving window for which we tested different widths (50, 100, 150, and 200 ms) and shifting intervals (with 50 ms steps and the maximum duration equal to the width of the window) in order to identify the configuration with the best performance. We computed the average of the data within the moving window and used these averaged normalized distances to train a machine learning classifier to estimate the bow section used, as described previously. We used the Classification Learner application of Matlab R2021a for the analysis.

## 3. Results

### 3.1. Measuring Bow–Violin Orientation

**Figure 7:**
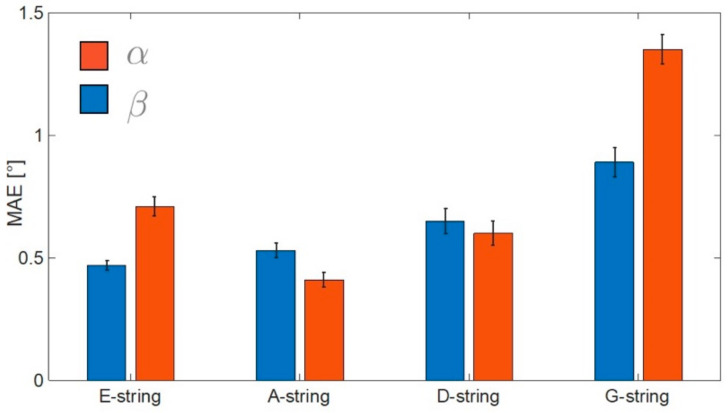
Mean absolute error (MAE) computed on each string for both α and β angles. Bars represent the standard error.

Figure 7 reports the mean (boxes) and the standard error (bars) of the MAE obtained for the nine subjects involved in the bow–violin orientation test. The G-string shows the maximum MAE for both angles. In particular, we obtained an error equal to 1.4° for the α angle and 0.9° for the β angle. In all the conditions tested, the MAE remained below 2° (Figure 7).

**Figure 8:**
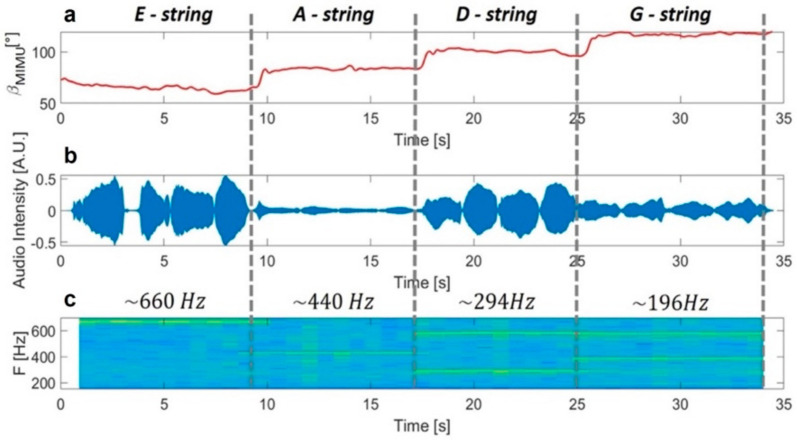
From the top: plot (**a**), 𝛽 angle measured by Xsens; plot (**b**), audio track; plot (**c**), time–frequency analysis.

**Figure 9:**
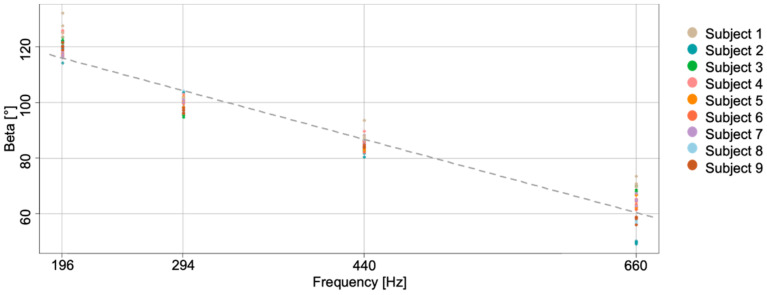
The plot shows the *β* angle averaged in each stroke vs. the corresponding string frequency. Data from each subject are identified with a different color. The dashed gray line represents the overall regression line ignoring the subject variable.

We further assessed whether the information about bow orientation can be exploited to provide students with additional feedback on the effective string played. In particular, we performed a correlation analysis between the string played and the bow tilt to determine whether the β angle can be exploited to derive information about the string played. The upper box of Figure 8 reports the β angle measured by MIMUs during the task for one representative subject. The second plot represents the audio track recorded during the exercise, which clearly shows how the subject performed the four strokes for each string (tip–frog and frog–tip two times). The last plot represents the time–frequency analysis of the audio track, and it shows the frequency characterizing each string: about 660 Hz for the E-string, about 440 Hz for the A-string, about 294 Hz for the D-string, and about 196 Hz for the G-string (see Supplementary Materials File S1). In particular, for the last two strings, it is also possible to visualize the second harmonics (D-string and G-string) and the third one (only G-string). We identified the string played, thanks to this plot, as subjects were requested to perform an open-string exercise, and thus, we could match the string with frequency. Figure 9 reports the β angle (dependent variable) vs. the frequency of the string played (independent variable), as measured for all subjects. We verified the assumptions for a repeated measures correlation analysis [19] and tested whether there was any correlation between the β angle and the frequency of the string played. The repeated measures correlation confirmed a high negative correlation between the tilt angle and the string frequency ($r\left(134\right)=-0.973,\;95\%\;\mathrm{CI}\;\left[-0.981,-0.962\right],\;p<0.001$).

### 3.2. Estimating the Bow Section

**Figure 10:**
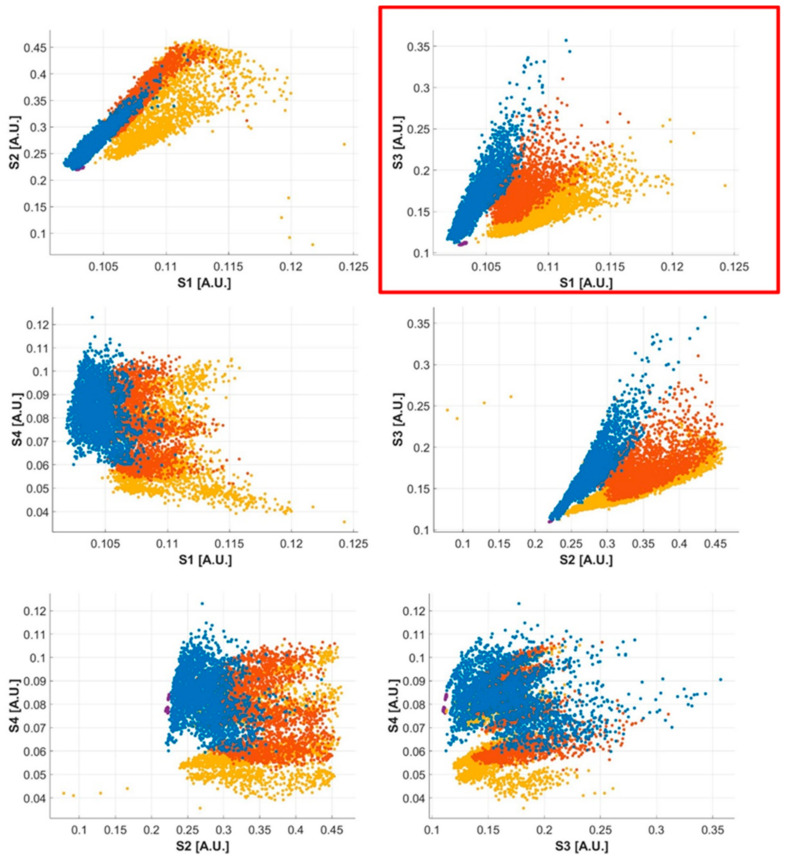
Clusters of points for all the possible combinations of sensor couples: S1–S2, S1–S3, S1–S4, S2–S3, S2–S4, and S3–S4. The S1–S3 couple produced well-defined clusters of points for the four investigated conditions. Lower section (blue), middle section (orange), upper section (yellow), and bow raised (violet).

Data from the four optical proximity sensors were normalized with respect to their full scale. Exploratory data analysis was performed to identify any possible simplification strategy in order to reduce the hardware complexity and computational effort. Considering all the possible combinations of sensor couples (Figure 10), we observed that the S1–S3 couple produces well-defined clusters of points for the four investigated conditions: lower section, middle section, upper section, and bow raised. For this reason, we focused on the data gathered from these two sensors. Data were split into moving windows. For each window, the normalized distance measured was averaged and used to train the two classifiers considered. The performance of the algorithms was evaluated for all the window widths and for the shifting intervals considered, with a hold-out method, considering 25% of the data as the test data set.

**Figure 11:**
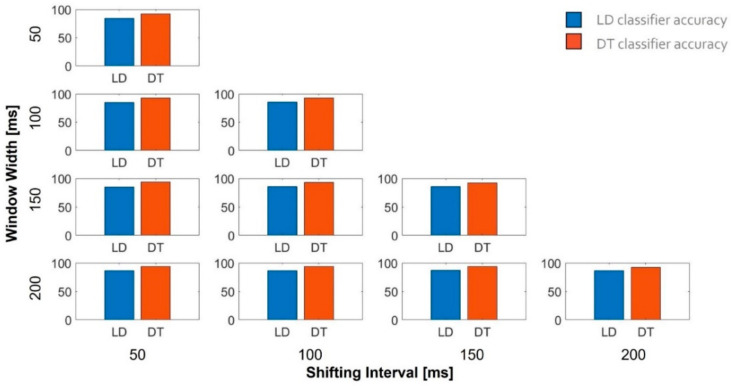
Accuracy of the two classifiers estimated for different combinations of window width and shifting interval.

Figure 11 shows the accuracy of the two classifiers when trained using different combinations of window width and shifting intervals. In all conditions, the DT classifier outperformed the LD classifier. The maximum accuracy (94.2%) was obtained for the DT classifier when trained with a window width of 200 ms and a shifting interval of 150 ms. These settings brought about an overlapping between two consecutives windows of 50 ms and provided students with feedback every 150 ms (i.e., with a frequency of 8 Hz).

## 4. Discussion

Learning to play the violin is a complex motor task that forces learners to perform uncomfortable movements in order to obtain a good sound. In this process, the hand–arm unit plays a crucial role both for technical and expressive purposes [20] and is therefore responsible for most of the musical outcomes. Therefore, it is of paramount importance to carefully monitor beginners’ motions and promptly correct them, when necessary. In this regard, although several technological systems have been developed ad hoc [4,5,6,7,8,9,10,11,12,13], only a few are specifically devoted to the training of beginners [5,13]. Additionally, the effectiveness of technology-enhanced practice in instrumental music teaching is still debated [21]. For instance, Tuuri and Koskela [22] observed that technology is often perceived as something unnatural and distant from how musicians live and contextually develop experience. In this respect, Leman and Nijs [21] underlined the importance of considering the cognitive workload the learners face to process information: good technology-enhanced practice should make it easier to process this information. Taking into account such issues, we proposed an instrument-centered interface directly embedded into the violin. Such a design has the advantage of not providing the students with additional information to manage; rather, it reinforces what is usually provided as feedback by the teacher, allowing students to correct themselves, even when the teacher is not present. Our system is based on the use of magneto-inertial measurement units and optical proximity sensors coupled with a classification algorithm to monitor the orientation of the bow with respect to the violin and the bow section used, respectively. These are two of the main parameters a beginner must learn to control when performing bowing-on-open-string exercises.

To validate the use of MIMUs to measure bow–violin orientation, we compared the reconstruction of the bow orientation obtained by commercial magneto-inertial sensors with the one obtained by an optical motion capture system, considered the gold standard. The results showed an orientation error less than 2°. A repeated measure analysis showed a high negative correlation between the tilt angle β and the string frequency ($r\left(134\right)=-0.973,\;\;95\%\;\mathrm{CI}\;\left[-0.981,-0.962\right],\;p<0.001$). Taken together, these results justify the use of magneto-inertial sensors to help beginners learn to control bow orientation with respect to the violin and the string. However, different sensors have been recently used to monitor bowing. For example, Di Tocco et al. [23] presented a wearable system based on piezoresistive sensors to monitor the wrist and elbow movements of a double-bass player. The system allowed the identification of string changes and bow strokes. However, although lightweight and adaptable, the system has to be mounted on a musician’s right arm, which may impact the ability of the musician to perform bowing movements in a natural way. In contrast, the interface we proposed is instrument centered, i.e., it is external to the user and therefore does not limit the user’s range of movements, nor can it be perceived as uncomfortable by the user. Moreover, our interface is not sensitive to different users’ anthropometries, thus increasing the reliability of the results. This also widens the potential usability of the system with both adults and children outside the lab in less structured and controlled contexts. Therefore, we chose MIMU technology, which does not require a structured environment and can be easily integrated into the instrument. Finally, since the interface proposed is deeply integrated into the instrument, it will not increase the level of discomfort for the musician.

We also exploited an instrument-centered approach in order to estimate the bow section in contact with the strings. We designed and developed an *I^2^C* network of four proximity sensors, which we distributed on the bow. While these sensors have been effectively used in the past [8], our experimental setup evidenced the need to address the problem of the large number of cables these sensors require. Our exploratory data analysis suggested that we can obtain reliable data from two out of the four sensors mounted on the bow, resulting in a considerable reduction in the hardware complexity. In fact, using only the S1–S3 couple and a decision tree classifier, we reached a classification accuracy of 94.2% with a window width of 200 ms and a shifting interval of 150 ms. This result needs to be confirmed with additional tests on a larger number of subjects. In fact, even if we used 75% of the data for training and the remaining 25% for testing, the data were not independent as they were obtained from the same subject. This single subject 75/25 train/test split increases the accuracy obtained compared to when these models are applied to other individuals. Despite this limitation, the results seem to suggest the possibility of providing students with online feedback at 8 Hz, compatible with the requirements of the application.

In the future, we plan to further reduce the hardware complexity by developing a modular architecture that will allow more efficient management of the cables and exploit wireless Bluetooth communication to exchange data with a remote laptop.

## 5. Conclusions

This feasibility study focuses on the possibility of developing a technology-enhanced tool to help violin beginners learn two fundamental skills of the bowing technique, the ability to control the relative orientation between the bow and the violin and the ability to properly use different sections of the bow. Among the different possible technological solutions available, we chose the magneto-inertial technology and optical proximity sensors using an instrument-centered approach. This allows the system to be used out of the lab in a minimally structured environment and reduces the computational workload of the student, improving the chances of the student learning the instrument.

The results of our preliminary validation confirmed that MIMUs are suitable to measure bow–violin orientation. The optical proximity sensors can be integrated in the bow to extract the section of the bow played. We verified the possibility of reducing the number of sensors with respect to previous studies [8,9] but additional tests are needed to confirm our preliminary results regarding the accuracy of classification. These preliminary results, if confirmed, could pave the way for a new class of instrumented tools for music, such as smart interfaces, to monitor human movement outside the lab, which could provide large data sets for research studies as well as real-time feedback to improve students’ behavior and learning curves.

## Figures and Tables

**Table 1:** The technology comparison table lists different technologies used to monitor bowing and provides information about the variables they monitor and the usability.

Technology		Variables to Be Monitored (Accuracy: High, Medium, Low)					Usability Parameters			
	Bow Section	Bow–Bridge Distance	Bow Velocity	Bow Acceleration	Bow Force	Bow Orientation	Invasiveness	Weight	Integration Difficulties	Cost
Resistive strip and capacitive coupling	+ (High)	+ (High)	d (Medium)	d (Medium)	− (N.A.)	− (N.A.)	Low	Low	High	Low
Optical sensors	+ (High)	− (N.A.)	− (N.A.)	− (N.A.)	d (Medium)	− (N.A.)	Low	Low	Medium	Low
Strain gauges	− (N.A.)	− (N.A.)	− (N.A.)	− (N.A.)	+ (High)	− (N.A.)	Low	Low	Low	Low
MEMS accelerometers	− (N.A.)	− (N.A.)	− (N.A.)	+ (High)	− (N.A.)	− (N.A.)	Low	Low	Low	Low
Optical marker systems	+ (High)	+ (Low)	d (Medium)	d (Medium)	− (N.A.)	+ (High)	High	Low	High	High
MIMU	− (N.A.)	− (N.A.)	− (N.A.)	+ (High)	− (N.A.)	+ (High)	Low	Low	Low	Low

(+) The variable can be directly measured; (−) the variable cannot be measured; and (d) the label can be derived from the row data. When the variable can be measured, the accuracy is reported in parentheses; N.A.: not applicable.

## Data Availability

The data presented in this study are available on request from the corresponding author. The data are not publicly available due to ethical restrictions.

## Supplementary Materials

The following are available online: at https://www.mdpi.com/article/10.3390/s21175817/s1, Video S1: Section Bowing Estimation-Pilot Test; at https://www.mdpi.com/article/10.3390/s21175817/s2, Audio File S1: bow–violin orientation test, audio track. Video S1 shows the expert musician performing strokes in the first third of the bow at slow tempo during the pilot trial. Audio File S1 is the audio recording corresponding to data reported in Figure 8.

## References

[1] Gladwell M. (2008). Outliers.

[2] Helmholtz H.V. (1985). On the Sensation of Tone.

[3] Schelleng J.C. (1974). La fisica delle corde di violino. Sci. Am..

[4] Askenfelt A. (1988). Measurement of the Bowing Parameters in Violin Playing. J. Acoust. Soc. Am..

[5] Young D. (2002). The Hyperbow Controller: Real-Time Dynamics Measurement of Violin Performance. Proceedings of the 2002 Conference on New Interfaces for Musical Expression.

[6] Paradiso J.A., Gershenfeld N. (1997). Musical Applications of Electric Field Sensing. Comput. Music J..

[7] Demoucron M., Askenfelt A., Caussé R. (2009). Measuring Bow Force in Bowed String Performance: Theory and Implementation of a Bow Force Sensor. Acta Acust United Acust..

[8] Pardue L.S., Harte C., McPherson A.P. (2015). A Low-Cost Real-Time Tracking System for Violin. J. New Music Res..

[9] Pardue L.S. (2017). Violin Augmentation Techniques for Learning Assistance. Ph.D. Thesis.

[10] Rasamimanana N., Kaiser F., Bevilacqua F. (2009). Perspectives on Gesture–Sound Relationships Informed from Acoustic Instrument Studies. Org. Sound.

[11] Schoonderwaldt E., Sinclair S., Wanderley M.M. Why Do We Need 5-DOF Force Feedback? The Case of Violin Bowing. Proceedings of the 4th International Conference on Enactive Interfaces.

[12] Ancillao A., Savastano B., Galli M., Albertini G. (2017). Three Dimensional Motion Capture Applied to Violin Playing: A Study on Feasibility and Characterization of the Motor Strategy. Comput. Meth. Programs Biomed.

[13] Linden J., Schoonderwaldt E., Bird J., Johnson R. (2010). MusicJacket—Combining Motion Capture and Vibrotactile Feedback to Teach Violin Bowing. IEEE Trans. Instrum. Meas..

[14] Brooke J. (1996). SUS-A quick and dirty usability scale. Usability Evaluation in Industry.

[15] Curci A. (1980). Tecnica Fondamentale Del Violino.

[16] R Core Team (2021). R: A Language and Environment for Statistical Computing.

[17] Matlab Choose Classifier Options. https://it.mathworks.com/help/stats/choose-a-classifier.html.

[18] Safavian S.R., Landgrebe D. (1991). A Survey of Decision Tree Classifier Methodology. IEEE Trans. Syst. Man Cybern..

[19] Bakdash J.Z., Marusich L.R. (2017). Repeated Measures Correlation. Front. Psychol..

[20] Leman M., Nijs L., Di Stefano N., Bertolaso M., Di Stefano N. (2017). On the Role of the Hand in the Expression of Music. The Hand Studies in Applied Philosophy, Epistemology and Rational Ethics.

[21] Leman M., Nijs L. (2017). Cognition and technology for instrumental music learning. The Routledge Companion to Music, Technology, and Education.

[22] Tuuri K., Koskela O. (2020). Understanding Human–Technology Relations Within Technologization and Appification of Musicality. Front. Psychol..

[23] Di Tocco J., Massaroni C., Di Stefano N., Formica D., Schena E. Wearable System Based on Piezoresistive Sensors for Monitoring Bowing Technique in Musicians. Proceedings of the 2019 IEEE SENSORS.

